# The Effect of Progestins on Cytokine Production in the Peripheral Blood Mononuclear Cells of Menopausal Women and Their Luminol-Dependent Chemiluminescence

**DOI:** 10.3390/molecules28114354

**Published:** 2023-05-26

**Authors:** Tatiana I. Pavlik, Nikolay L. Shimanovsky, Olga A. Zemlyanaya, Tatiana A. Fedotcheva

**Affiliations:** Science Research Laboratory of Molecular Pharmacology, Medical Biological Faculty, Pirogov Russian National Research Medical University, Ministry of Health of the Russian Federation, Ostrovityanova St. 1, 117997 Moscow, Russia

**Keywords:** progesterone, MPA, PBMC, IL-6, IL-1β, TNFα, TGFβ, MTT, luminol-induced chemiluminescence

## Abstract

Steroid hormones are the key regulators of inflammatory and autoimmune processes. The role of steroid hormones is mostly inhibitory in these processes. The expression of IL-6, TNFα, and IL-1β, as markers of inflammation, and TGFβ, as a marker of fibrosis, could be useful tools to predict the response of an individual’s immune system to the different progestins suitable for the treatment of menopausal inflammatory disorders, including endometriosis. In this study, the progestins P4 and MPA, as well as the novel progestin gestobutanoyl (GB), which possess potent anti-inflammatory properties towards endometriosis, were studied at a fixed concentration of 10 µM. Their influence on the production of the above cytokines in PHA-stimulated peripheral blood mononuclear cells (PBMCs) during 24 h incubation was evaluated by ELISA. It was found that synthetic progestins stimulated the production of IL-1β, IL-6, and TNFα and inhibited TGFβ production, while P4 inhibited IL-6 (33% inhibition) and did not influence TGFβ production. In the MTT-viability test, P4 also decreased PHA-stimulated PBMC viability by 28% during 24 h incubation, but MPA and GB did not have any inhibitory or stimulatory effects. The luminol-dependent chemiluminescence (LDC) assay revealed the anti-inflammatory and antioxidant properties of all the tested progestins, as well as some other steroid hormones and their antagonists: cortisol, dexamethasone, testosterone, estradiol, cyproterone, and tamoxifen. Of these, tamoxifen showed the most pronounced effect on the oxidation capacity of PBMC but not on that of dexamethasone, as was expected. Collectively, these data demonstrate that PBMCs from menopausal women respond differently to P4 and synthetic progestins, most likely due to distinct actions via various steroid receptors. It is not only the progestin affinity to nuclear progesterone receptors (PR), androgen receptors, glucocorticoid receptors, or estrogen receptors that is important for the immune response, but also the membrane PR or other nongenomic structures in immune cells.

## 1. Introduction

The search for highly active progestins for the treatment of hormone-dependent tumors and endometriosis is an urgent task, since at present there are very few such molecules, and the mechanisms of their antitumor and anti-inflammatory activities are not well understood. At an appropriate dosage and regimen of administration, progestins could be beneficial drugs for the treatment of menopausal disorders, accompanied by chronic inflammatory or fibrosis processes. The choice of progestin for the treatment of menopausal disorders and for HRT is an important consideration.

Medroxyprogesterone acetate and megestrole acetate—pregnane progestins—have indications for use in anticancer therapy and HRT [1,2]. The novel pregnane progestin gestobutanoyl has previously been studied in animals as a promising progestin for the treatment of endometrial hyperplasia and endometriosis and for maintaining pregnancy [3]. In models with endometriosis, it was not inferior to dienogest in therapeutic activity as an effective medicine in the treatment of endometriosis [4]. Its antitumor activity towards hormone-dependent tumors, such as cervical cancer in vivo and on different cancerous cell lines in vitro, has been established [5].

**Figure 1:**
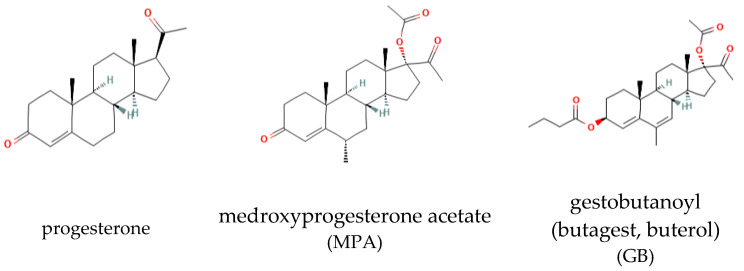
Structure of the progestins.

The structural formulas of the studied progestins are shown in Figure 1.

In this study, we use the blood of menopausal women aged 60–65 years (average of 61.8 ± 2.1 years) to determine the profile of the influence of progestins on cytokine production. It was shown earlier that P4 increased LPS-stimulated IL-6 and TNFα production in healthy young women and decreased IL-6 and TNFα production in a cancerous mononuclear cell line [6]. Controversial results were obtained from women using combined oral contraceptives: they had lower levels of pro-inflammatory cytokines and the treatment of their PBMCs with E2 and P4 decreased the levels of lymphoproliferation and pro-inflammatory cytokines (IFN-, IL-12p70, and TNFα) [7]. In line with this, in a culture of PBMCs from women with chronic inflammatory disease, such as unexplained recurrent spontaneous miscarriage (uRSM), dydrogesterone and progesterone up-regulated IL-6 but down-regulated IL-17A and IL-23 [8]. A decrease in ovarian function during menopause is accompanied by an increase in TNFα, IL-1β, and IL-6 [9], which may indicate a protective role of female sex hormones against this cytokine action. This is also supported by data showing higher levels of TNFα and IL-1β in men with hepatitis C than in premenopausal women with the same pathology [10].

The debatable question is which hormone, P4 or E2, causes stronger anti-inflammatory and immunomodulatory effects, as well as their dose dependence. Straub proposed that 17β-Estradiol exhibits a biphasic dose–response relationship with TNFα, IL-1, and IFN, in which higher doses suppress production and lower doses increase cytokine production [11]. Yuan showed that oxidative stress-stimulated cytokine expression was attenuated by 0.1 µM E2 and augmented by 0.1 µM progesterone in a dose-dependent manner, without any significant difference between males and females [10].

The data are inconsistent regarding the effect of steroid hormones, particularly progestins, on cytokine production by mononuclear cells from menopausal women, and there is a lack of data about their influence on the viability of PBMCs and their oxidative capacity. MPA and P4 are frequently used drugs in HRT and anticancer therapy, but their mechanism of action have not been clearly identified. The aim of the study is to investigate the effect of P4 and synthetic progestins (including the new progestin GB) on IL-1, IL-6, TNFα, and TGFβ secretion after PHA stimulation in PBMCs from menopausal women. To determine the possible anti-inflammatory and immunomodulatory action of GB as a potent drug for HRT and endometriosis therapy, we compare the influence of GB on the viability and oxidative capacity of PBMCs with other steroid hormones with known anti-inflammatory action (the glucocorticoids dexamethasone and cortisol) and with hormones and their antagonists, whose anti-inflammatory action is controversial (such as estradiol, testosterone, cyproterone, and tamoxifen).

## 2. Results

### 2.1. Progestin Influence on the Viability of PBMCs

**Figure 2:**
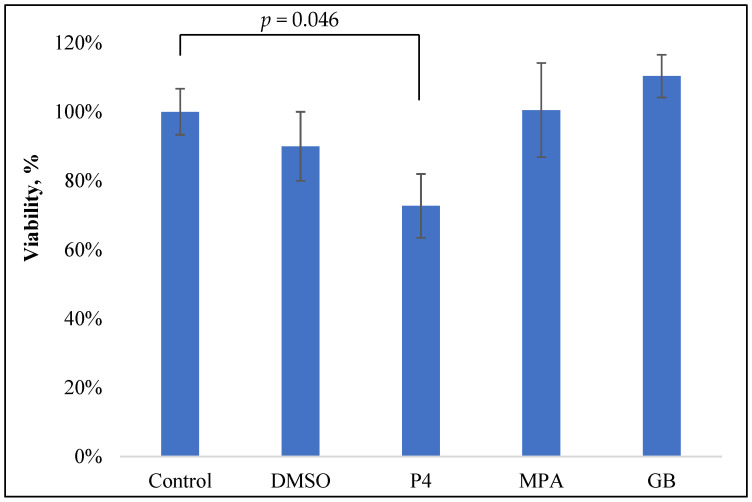
MTT test of the PBMC viability after 24 h incubation with PHA (0.1 mg/mL) and progestins (10^−5^ M).

For the estimation of the influence of progestins on the viability of the PBMCs, freshly isolated PBMCs were incubated with PHA and steroids for 24 h and colored with MTT, as previously described [12]. The control wells contained the solvent for the studied steroids (DMSO) in equivalent concentrations. The data are shown in Figure 2.

Only P4 slightly inhibited the viability of PBMCs: 28% inhibition at 10^−5^ M (*p* = 0.047). Neither GB nor MPA, nor the control solvent DMSO, influenced PBMC viability.

The obtained data about P4 inhibitory activity are in good accordance with previously obtained results on E. coli-stimulated horse PBMCs: P4 (1 µg/mL or 0.3 µM) inhibited PBMC viability by approximately 30% [13].

### 2.2. Influence of Progestins on the PBMC Oxidant Capacity

PBMC oxidant capacity, or the so-called leukocyte coping capacity (LCC), reflects the PBMC response to multiple stress signals and is associated with its immune function [14]. A constantly low LCC directly reflects increased stress levels and a reduced (innate) immune function [14]. If the functionally active PBMCs are exposed to NSAIDs (non-steroidal anti-inflammatory drugs), LCC starts to decrease [15]. The chemiluminescence curves can be indicators of the anti-inflammatory action of a novel drug.

In this study, freshly obtained PBMCs from the blood of menopausal women were resuspended in PBS and were immediately measured for LCC. The substances were added 1 min before the chemiluminescence measurement, so the subsequent effect is thought to be nongenomic. For the controls, an equal amount of the solvent DMSO was added. DMSO is also a well-known anti-inflammatory drug and penetration enhancer for the topical administration of other NSAIDs, available as RIMSO-50^®^ (dimethyl sulfoxide) [16]. It has been indicated for the symptomatic relief of patients with interstitial cystitis by direct intravesical instillation [17]. In the Russian Federation, DMSO is available as Dimexide 25% gel or concentrate for the preparation of solutions for external use and is indicated for the complex treatment of rheumatoid arthritis, ankylosing spondylitis, and other inflammatory diseases [18].

**Figure 3:**
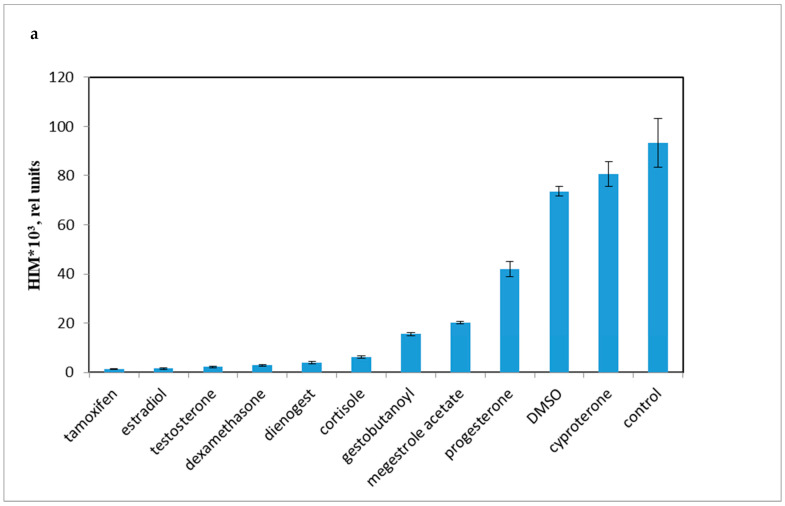

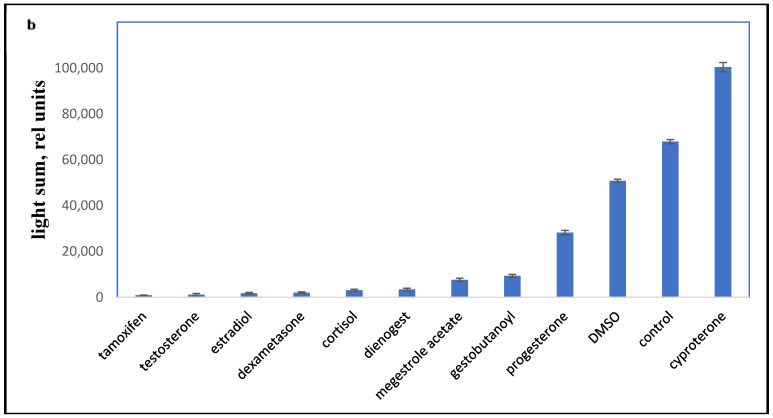
PBMC oxidant capacity after drug treatment. (**a**) the average chemiluminescence intensity maximum (HIM) of PBMC; (**b**) the average chemiluminescence light sum of PBMC. Note: HIM was recorded as the maximum of the light sum of the luminol-dependent chemiluminescence measured for 10 min immediately after the drug was added to the PBMCs. All steroids and tamoxifen were tested at 10^−5^ M; DMSO was 0.1%. BaSO_4_ was added 10 s before the tested drugs.

Accordingly, it was necessary to compare the influence of progestins on LCC with DMSO, as the effect of DMSO on LCC and PBMC viability have not been previously established. As shown in Figure 3a, progestins and DMSO caused a decrease in chemiluminescence intensity (HI). The average chemiluminescence intensity maximum (HIM) was calculated after a 10 min recording of the chemiluminescence. The light sum values are shown in Figure 3b. Rapid, nongenomic effects were detected as steroid substances were added immediately prior to the 10 min chemiluminescence recording.

All the tested compounds inhibited the oxidant activity of PBMCs, except for cyproterone acetate, which is essentially an antiandrogen.

The most effective down-regulator of LCC was the antiestrogen tamoxifen. Conversely, cyproterone, a progestin with antiandrogen activity, had some stimulatory effect on LCC (*p* < 0.001). Synthetic progestins were less effective down-regulators than glucocorticoids, but were more potent than P4 and DMSO.

The activities of enzymes such as SOD, GST, GPX, and NOS contribute to chemiluminescence intensity, and the activity of these enzymes is inhibited by P4 [19]. In patients with autoimmune chronic diseases (for example, rheumatoid arthritis), the PBMC oxidative activity is usually reduced, which can be explained by the decreased activity of these antioxidant enzymes.

NSAIDs traditionally reduce the oxidant activity of macrophages [15,20]. Since progestins inhibit NOS and GST [19], this can be the reason, at least partially, for their inhibitory effect on LCC.

### 2.3. Influence of Progestins on the Production of Cytokines in PBMCs

There are data on the inhibition of the synthesis of TGFβ, TNFα, IL-6, and IL-1β by progestins in cancer cells [6]. This effect can be achieved through the binding of progestins to nuclear glucocorticoid receptors or through their direct stimulation of normal immune cells [6]. The data are contradictory, since the production of cytokines depends on the type of mononuclear cells, their localization, the stage of inflammation (acute or chronic), and the presence of an autoimmune process.

The cytokine profile in women with preterm birth (PTB) and recurrent miscarriages has been particularly widely studied. The PBMCs of these women have elevated levels of TNFα, IL-6, and IL-1β [21,22,23]. Elevated levels of IL-6 have also been demonstrated in women with breast cancer and can be one of the main reasons for anorexia/cachexia. It is assumed that the anticachetic effect of MPA or MGA is due to the inhibition of IL-6, IL-1β, and TNFα production [24,25].

Some studies [6] have described the multidirectional effects of P4 and progestins in PBMCs: P4 (50 μM) increased the mRNA of IL-1β, TNFα, and IL-6 cytokines; in monocyte cell-cultured THP-1, similarly to PBMCs, the expression of IL-1β and TNFα mRNA was increased, and in Jurkat leukemic T cells, the expression of IL-2 and TNFα mRNA decreased [6].

In this study, we explored the influence of P4 and its analogues on cytokine production in a model of PBMCs from menopausal women, with the aim of identifying the appropriate progestins for HRT and those with potential anti-inflammatory action.

**Figure 4:**
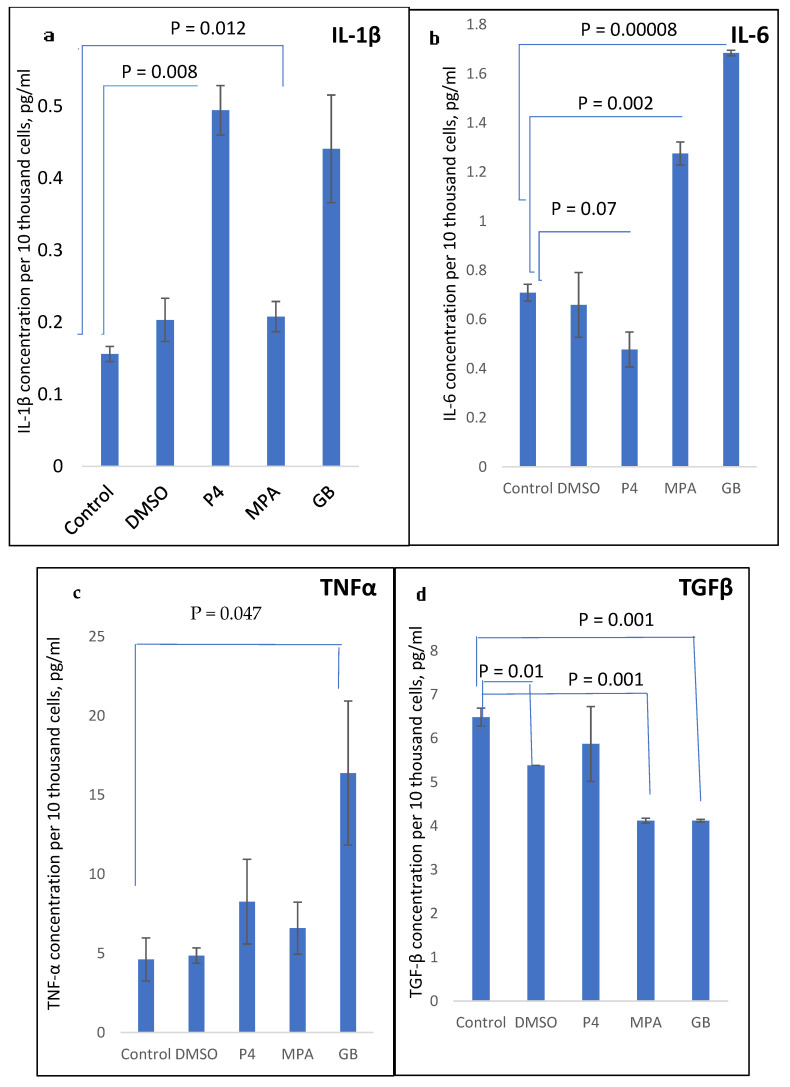
(**a**) IL-1β, (**b**) IL-6, (**c**) TNFα, and (**d**) TGFβ concentration in PBMC supernatant, measured after 24 h incubation with PHA and progestins.

The influence of different progestins on the synthesis of pro-inflammatory cytokines IL-1β, TNFα, and IL-6 in PHA-stimulated PBMCs from healthy menopausal women is shown in Figure 4a–c. TGFβ synthesis upon treatment with progestins is shown in Figure 4d.

As shown in Figure 4, the progestins MPA and GB act in the same manner, demonstrating a stimulatory pro-inflammatory effect on IL-6, TNFα, and IL-1β production and inhibitory effect on TGFβ production. The influence of GB was the most pronounced towards IL-6 and TNFα stimulation. P4 demonstrated a stimulatory effect on TNFα and IL-1β production and inhibitory effect on IL-6 production, and did not influence TGFβ production.

Therefore, there is a difference between the action of P4 and its synthetic analogues. IL-6, unexpectedly, was down-regulated by P4 and up-regulated by MPA and GB.

Progestins act on immune cells via mPR and have both pro- and anti-inflammatory effects, depending on the phenotype of these cells. The action of synthetic progestins compared to P4 may depend on different affinities for RP, both for nuclear and membrane ones, that is, it depends on their chemical structure and binding characteristics with receptors. The data obtained are important for understanding the complex regulation of the immune system by progestins, which depends on the type of immune cells and the individual characteristics of the immune system, which, in turn, depends on age, kind of disease, the phase of the menstrual cycle, and, accordingly, the initial level of progesterone and estradiol in women’s blood. It has recently been established that polymorphisms (SNPs) of progesterone, estradiol, and glucocorticoid receptor genes also affect acquired and trained immunity [26,27].

## 3. Discussion

Progesterone is known as an anti-inflammatory and immunosuppressive hormone that achieves these effects via nuclear and membrane PR on host and immune cells [28]. P4 can also act via GR, to which P4 has affinity, as well as some synthetic progestins, such as MPA [29]. The influence of P4 and progestins on the viability and production of cytokines by PBMCs has not been thoroughly described and understood; to date, only controversial results have been obtained. It is known that P4 and MPA at 10 μM did not influence PBMC viability from healthy young women [30]. In our study, MPA and GB did not influence the PBMC viability of menopausal women, but P4 slightly inhibited it at a concentration of 10 μM. In this experiment, the effect of P4 differed from that of MPA and GB, which tended to increase PBMC viability.

The action of P4 differed from that of MPA and GB on PHA-stimulated cytokine production. Previous studies on PBMCs have demonstrated the specific, nuclear-receptor-dependent and -independent action of P4 on cytokine production, which is diminished by the PR antagonist RU486 [31]. In the same mode of action, Miller showed that P4 can specifically down-regulate TNFα mRNA and protein production in activated macrophages by NF-kappa B inhibition, but E2 did not influence it [32].

Since many progestins bind not only to progesterone receptors, but also to glucocorticoid, androgen, and mineralocorticoid receptors and possibly to estrogen receptors, it is plausible that synthetic progestins exert therapeutic actions as well as side-effects via some of these receptors [33].

MPA and GB, as synthetic analogues of P4 that belong to pregnane progestins, have more affinity to PR than to GR and AR. At the same time, the specific progestin activity of GB is 65 times higher than the progestin activity of P4, which was estimated in the classical Clauberg–McPfail test on rabbits [34]. Possibly, GB pharmacological activity depends not only on its affinity to PR, but also on the affinity to the membrane PR. This can achieve a rapid nongenomic response. The rapid (1 h incubation) nongenomic anti-inflammatory effect of GB has been demonstrated in the previous studies on rat skin fibroblasts, where GB and glucocorticoids increased the redox system parameters to a great extent [35].

The analysis of the binding of GB to nuclear PRs has been carried out on several objects—tumor cell culture [36], rabbit and rat wombs [34], and mononuclear fractions of women [37]. Data on the binding of GB to RP in comparison with some known progestins are presented in Table 1.

Table 1 shows that the affinity of GB to the nuclear receptors is significantly lower than that of MPA, progesterone, and levonorgestrel.

RBA for GB binding to AR and GR was 0.22% in comparison with dihydrotestosterone and 0.14% in comparison with dexamethasone [34]. The extremely low ability of GB to bind to AR and GR was confirmed in animal studies: gestobutanoyl at a dose of 25 mg/kg had no androgenic and glucocorticoid effects [41].

The most pronounced immunomodulatory action of GB is not connected with GR. This effect does not contribute to AR and does not fully contribute to nuclear PR, as the affinity to nPR is lower than for the other studied progestins (Table 1). The progestins medroxyprogesterone acetate and norethisterone acetate caused a different effect on pro-inflammatory cytokine genes, depending on the cell type obtained from the female genital tract [42]. The differential action of sex steroid hormones on cytokine production could be the reason for the almost equal clinical effectiveness of the antiprogestin mifepristone and some progestins in the treatment of chronic processes, such as uterine fibroid.

In our study, MPA and GB did not influence PHA-stimulated PBMC viability, and only P4 inhibited cell viability by 30% during 24 h incubation. Conversely, MPA and GB inhibited PBMC oxidant capacity to a greater extent than P4. Interestingly, all tested hormones inhibited PBMC oxidant capacity. This effect may be due to the rapid nongenomic effects of steroid hormones, which can be achieved through membrane progesterone receptors but not through nuclear PR, as the time of incubation did not exceed 1 min.

The PBMC oxidant capacity, in our study, was measured by the luminol-dependent chemiluminescence method. This method is a very valuable indicator that allows the evaluation of important types of drug activity, such as antioxidant, anti-inflammatory, and anti-allergic, as well as the evaluation of how the hydrophilic and hydrophobic components could affect the peroxidation of the lipid membrane of cells, the formation of radicals in mitochondria, and the effect of oxidative stress on the body in systemic vasculitis, type 2 diabetes mellitus, rheumatoid arthritis, and Parkinson’s disease [43,44].

The possibility of P4 and progestins to decrease PBMC oxidant capacity is an important benefit in their application to treat chronic inflammatory diseases, which are accompanied by an elevated PBMC oxidant capacity.

MPA and GB also significantly influenced cytokine production by PBMCs from menopausal women. At the studied concentration (10^−5^ M), this effect was unexpected: pro-inflammatory IL-1b, IL-6, and TNFα were elevated, which demands discussion and concretization as to, particularly, the fundamental characterization of the role of IL-6, IL-1β, and TNFα in women’s health over their entire lives.

Firstly, the profile of cytokine production strongly depends on the type and phenotype of the cell. As an example, THP-1 and U937 are two widely used, established human monocytic cell lines considered to be capable of mimicking some responses of primary monocytes and macrophages. They have distinct biological responses to the same stimuli, which makes them not ‘interchangeable’ as in vitro models of macrophage precursor cells. Under standardized PMA-induced differentiation conditions, THP-1-derived macrophages are more responsive to pro-inflammatory (M1) stimuli, whereas U937-derived macrophages are more responsive to M2 stimuli [45].

The opposite effect of P4 on cytokine production in cancerous and noncancerous cells has been demonstrated in studies: P4 mostly down-regulated IL-1β, IL-6, and TNFα in cancer cell lines and tissues [25,46]. MPA also decreased these cytokines in vitro in PBMCs from cancer patients [47]. MPA reduced IL-6 and TNFα in the PBMCs of young (19–40 years old) healthy women in [48], but P4 and E2 did not have a significant effect on cytokine production. In the above-mentioned studies, incubation with steroids occurred 24 h before PHA stimulation and the subsequent PHA stimulation took place for 24 h. In our study, PHA and steroids were added simultaneously to the PBMCs for 24 h. The incubation period as well as type of cells could be the reasons for these differences.

Secondly, the differences between pro- and anti-inflammatory cytokines is not very evident, especially considering their role in acute and chronic inflammation conditions. In acute inflammation processes, cytokines, such as IL-6, IL-1β, and IL-2, have a protective role. If chronic inflammatory and autoimmune processes occur, they can damage host cells.

It may not be entirely true to state unequivocally that IL-6 is a pro-inflammatory cytokine: there are at least two types of IL-6 receptors—IL-6 receptor (IL-6R) and soluble IL-6 receptor (sIL-6R)—which promote anti- and pro-inflammatory outcomes [49].

Progestins with a high affinity for GR have immunosuppressive effects inherent to GC, such as the immunosuppressive drug dexamethasone, which inhibits IL-6 and TNFα [50]. For example, MPA is a potent agonist for endogenous GR-regulated IL-6 genes in PBMCs [51]. Progestins with a high affinity for GR can either induce IL-6 synthesis or inhibit it.

Thirdly, cytokine production also depends on age and hormonal status, so the in vivo and in vitro effects of progestins can vary greatly. The effect of P4 on TGFβ, TNFα, IL-6, and IL-1β production in the PBMCs of healthy women have been previously studied, but there are no unidirectional results, as was mentioned above. At present, it is becoming clear that this effect depends on a number of factors, such as time after PHA stimulation, the type of immune stimulant (PHA or LPS), and the kind of progestin.

Fourth, the place of the production of mononuclear cells also affects their ability to synthesize cytokines.

The action of P4 on the PBMCs depends on the location of the PBMCs. Thus, in PBMCs isolated from human placental blood, after stimulation with LPS, P4 (0.01, 0.1, or 1.0 µM) significantly decreased the production of IL-1β, TNFα, IL-6, IL-8, and MIP-1α [52,53].

In an earlier prospective study, the production of TNFα and IL-1β by LPS-stimulated monocytes in the blood of men and postmenopausal women in vitro was not influenced by incubation with different concentrations of 17beta-estradiol or progesterone [54]. In 2004, another group showed that progesterone increased TNFα secretion in activated monocytes but decreased IL-6 [55].

As shown in our study, in the PBMCs of menopausal women, IL-6 was stimulated by synthetic progestins but not by P4, which is partially consistent with the previously obtained data of IL-6 increasing in PBMCs under the influence of P4 and dydrogesterone in women with recurrent miscarriages [8]. This seems paradoxical, since progesterone and dydrogesterone are usually used as a standard therapy for maintaining pregnancy in women with recurrent miscarriages [3], and one of the mechanisms of pathogenesis of their therapeutic effect is a decrease in circulating IL-6.

In predicting the effect of progestins in vivo, it can be assumed that a long exposure time is required to suppress the production of cytokines. To achieve a progesterone concentration on the order of micromoles, repeated administration of the hormone is required. Progesterone is important for the maintenance of pregnancy, where it is used repeatedly, creating a high concentration in vivo on the order of micromoles. The ways in which P4 exerts anti-inflammatory and immunomodulatory effects in maintaining maternal–fetal tolerance has remained unexplored. In a recent study, the authors concluded that PTP is associated with the activation of an inflammatory pathway leading to the induction of PR-A by B cells. The increased expression of PR-A in B cells has been associated with elevated maternal plasma concentrations of IL-6, IL-21, and TNFα in sPTB patients. Reduced progesterone levels in these women may lead to an increased expression of their receptors. This can further provoke inflammation, lead to a disruption of the mother’s tolerance for the fetus, and induce labor [56]. As indicated, to maintain a pregnancy, progesterone or dydrogesterone, probably through PIBF, reduce the production of IL-6 and prevent preterm birth [57].

In healthy women, the activation of the soluble IL-6 receptor coincides with the implantation window, and when TNFα levels rise, menstrual bleeding is initiated, while progesterone levels fall. Therefore, in healthy women, cyclically changing levels of progesterone and estradiol regulate the production of cytokines [58].

In the menopausal period, when the concentration of P4 and E2 is reduced, the exogenous administration of these hormones as part of HRT can have a positive effect by reducing the production of pro-inflammatory cytokines. Anti-TGFβ therapy is also desirable for menopausal women, as it reduces fibrosis processes.

In our study, P4 reduced IL-6 and TGFβ production in the PBMCs of menopausal women, while MPA and GB stimulated IL-6 and TNFα.

These complex relationships between sex hormones and the immune system, and the multidirectional effects of the hormones on cytokine production, require further study. Androgens, such as progestins, have different effects on cytokine production by immune cells, depending on the expression profile of the sex steroid receptors. Androgens and P4 mostly promote immunosuppressive or immunomodulatory effects, whereas estrogens enhance humoral immunity in both men and women [59]. Changes in the concentration of sex hormones during different periods in the lives of men and women strongly affect the immune status and response to hormone therapy. The level of sex hormones in the blood is strongly influenced by the microbiome. Male and female adults with excessive testosterone or estradiol concentrations, respectively, demonstrate a more diverse gut microbiome [60]. Recently, the gut microbiome composition has been shown to be an important regulator of sex hormone levels and immune system reactivity [60]. The gut microbiota can individually change cytokine production in response to exposure to exogenous hormones.

Synthetic progestins can differ from P4 in their action on immune cells, as was shown in our study. P4 itself can cause opposite effects on cytokine production. P4 acts differentially on TNFα production in PBMCs, possibly due to the age and hormonal profile of the woman: TNFα was not influenced at 10 µM of P4 in the PBMCs of menopausal women (which tended to increase), but it was down-regulated in the PBMCs of young pregnant women with the same P4 concentration in another study [61].

The complexity also lies in modeling an experiment to study the effect of hormones on the production of cytokines. Due to the impossibility of the long-term incubation of PBMCs, it is difficult to predict the delayed effects of hormones. P4, at the high concentration in our work, after 24 h incubation stimulated the production of TNFα and IL-1beta, but suppressed IL-6. If so, the induction of IL-6 during ovulation, when the concentration of P4 in the woman’s blood is maximum, can be explained. Ovulation is accompanied by an increase in the basal body temperature and an inflammatory response in the ovaries, and low IL-6 levels are found in chronic anovulation [62]. A decrease in IL-6 and TNFα can also be the reason for the threat of preterm birth. Therefore, the administration of P4 or dydrogesterone for quite a long time during pregnancy is justified.

The effect of P4 on the production of cytokines by tumor cells deserves special attention. IL-6 level is elevated in cancer, and the progestins MA and MPA decrease it, which shows their anti-cachectic effect, since it is assumed that features of CACS (cancer-related anorexia/cachexia syndrome) can be reproduced in vivo by the chronic administration of pro-inflammatory cytokines, including interleukin-I (IL-I), IL-6, and TNFα, either alone or in combination [24].

TGFβ inhibition by all the studied progestins is an expected and promising result, since, for menopausal women, anti-TGFβ therapy is desirable, due to the main TGFβ effect—fibrosis. The down-regulation of fibrosis may be a promising strategy in the treatment of uterine fibroids and surgery consequences. It has been already shown that preoperative long-term MPA treatment significantly decreases primary adhesion formation after surgery in rats [63].

One of the aims of this study was to evaluate the influence of the novel progestin gestobutanoyl on the viability, cytokine production, and oxidant activity of PBMCs in menopausal women. In this paper, we showed that, although GB inhibits the oxidative activity of macrophages, it also stimulates the production of pro-inflammatory cytokines, which may be useful in the fight against acute infections, but undesirable in chronic diseases, such as recurrent miscarriage, endometrial hyperplasia, or cancer. The anti-TGFβ action of GB and other progestins, studied in this work, demonstrates their importance in the treatment of endometriosis and cancer.

The choice of progestins in endocrine therapy may have implications for women with a risk of susceptibility to infections. This choice should be based on the knowledge of the nature of their action on the genes involved in inflammation and immune functions.

## 4. Materials and Methods

### 4.1. Drug Preparation

PHA: A total of 1 mg of PHA (“PanEco”, Moscow, Russia) was diluted in 1 mL of DMEM (“PanEco”, Moscow, Russia) with amphotericin B (“Biokhimik”, Saransk, Russia) and gentamicin (“PanEco”, Moscow, Russia). A total of 50 µL of this solution was added to a sample with PBMCs to a total volume of 500 µL, to achieve the final concentration of 0.1 mg/mL.

Steroids were purchased from “Sigma”, Sigma-Aldrich, St. Louis, MI, USA. Gestobutanoyl (17α-acetoxy-3β-butanoyloxy-6-methylpregna-4,6-dien-20-one (GB), of purity higher than ≥99.0% (HPLC), was provided by the Research Laboratory of Molecular Pharmacology, Moscow. Steroids were diluted in DMSO to a concentration 10 mM, and then diluted to a concentration of 0.1 mM in DMEM + Amphotericin B + Gentamicin. For the viability assay, 20 μL of these solutions was added to 180 μL of samples with PBMCs. In the respective control wells, 2 μL of DMSO was added, which corresponds to the DMSO concentration in the experimental samples with steroids.

### 4.2. Participants

The PBMCs were obtained from volunteers, which included staff members of the Pirogov Russian National Research Medical University, who were healthy women in the age range of 60–65 (n = 6). Informed consent was obtained from all included participants. Included participants had no medication with hormonal replacement therapy, contraceptives, or medication with NSAIDs. Women were non-smokers, with an average body mass index of 26.12 + 2.13, without serious comorbidities. The study was conducted in accordance with the Declaration of Helsinki, and approved by the Institutional Review Board of Pirogov, Russian National Research Medical University, N11/2022, 10 September 2022. PBMCs were obtained by the combination of centrifugation and sedimentation at 1 g based on sedimentation in a single-stage Ficoll density gradient [64]. The average composition of the fractions was as follows: T lymphocytes, 52.8 ± 3.5%; B lymphocytes, 5.7 ± 1.02%; and monocytes, 8.1 ± 0.8%, and were within the reference values.

### 4.3. MTT Assay

PBMC viability was assessed by the MTT assay. PBMCs were seeded in 96-well plates and were incubated in the presence of PHA (0.1 mg/mL) and progestins (10 µM) for 24 h. The DMSO concentration did not exceed 0.01%. The control wells contained the same volume of DMSO as the sample wells. At the end of the incubation, the medium in which the cells with the substances were cultivated was replaced with a DMEM medium without the addition of fetal calf serum, containing 5 mg/mL MTT, and the incubation continued for another 2 h in a CO_2_ incubator. After incubation, the medium with MTT was removed and 150 µL of DMSO was added to the wells to dissolve the formazan precipitate. The optical density of the samples was measured at a wavelength of 530 nm on a photometer “UNIPLAN” AIFR-01 (Pikon, Moscow, Russia). PBMC viability was expressed as a percentage relative to the values obtained for the control samples (without the tested compounds). The average values of the optical density in the control samples were taken as 100%.

### 4.4. LCC Measurement by the Luuminol-Dependent Chemiluminescence Assay

The stimulated luminol-dependent chemiluminescence (SLCL) was measured using a Lum-100 chemiluminometer, DiSoft, Russia. The data were evaluated using the PowerGraph 3.3 Professional software (DISoft, Moscow, Russia). The area under the chemiluminescence curve was estimated using the introduction of barium sulfate to the end of the kinetics (light sum) as well as the maximum amplitude of SLCL.

The chemiluminescence of the samples, measured in relative light units (RLU), was recorded for 10 min. The measurements were performed immediately after the blood samples were collected.

Various hormonal substances of different classes were used as reference drugs to assess the specificity of progestins: glucocorticoids (dexamethasone and cortisol), testosterone, estradiol, antiestrogen tamoxifen, pregnane progestins (MA and DG), and cyproterone acetate as progestin with antiandrogen action.

Initially, a control sample was prepared, consisting of 100 µL of PBMCs, adjusted to 750 µL with Hank’s solution (pH = 7.45), which was then subjected to incubation at 37 °C for 45 min. Next, the incubated sample was added to the cuvette of the chemiluminometer, and 150 µL of a luminol solution (Sigma-Aldrich, St. Louis, MI, USA) at a concentration of 4.5 mM was added (the final concentration in the sample was 0.56 mM). Spontaneous kinetics were recorded for 3 min, after which 300 µL of an activator of the oxidative activity of cells (a luminescence stimulator, barium sulfate, VIPS-MED Firm, Fryazino, Russia) was added at a concentration of 34.3 mM (final concentration in the sample was 8.6 mM), and the kinetics of the stimulated chemiluminescence were recorded for 10 min. The final sample volume was 1200 µL. The chemiluminescence measurement was carried out at 37 °C for 10 min. The light sum and HIM of the stimulated luminol-dependent chemiluminescence were measured immediately after the addition of the drugs to the fresh PBMCs.

### 4.5. ELISA

In this study, we used mitogen phytohemagglutinin (PHA-88/4, manufacturer PanEKO, Moscow, Russia) for PBMC stimulation. A total of 1 mg of PHA was diluted in 1 mL of DMEM medium (manufactured by “PanEKO”) + Amphotericin B (manufacturer (“Biokhimik”, Saransk, Russia) + Gentamicin (manufactured by «PanEKO», Russia). PHA solution (50 μL) was added to the samples with PBMCs and nutrient medium (450 µL DMEM medium + Amphotericin B + Gentamicin + 50 µL fetal bovine serum) to a total volume of 500 µL, to achieve a final PHA concentration of 0.1 mg/mL, and then incubated for 24 h in a CO_2_ incubator at 37.0 °C. After 24 h incubation with PHA, the drug substances were added, up to a final concentration 10 µM. TNFα, IL-1b, IL-6, and TGFβ antigen test kits were used to detect cytokine concentration, to perform ELISA on blood plasma samples according to the manufacturers’ instructions. The ELISA test systemd for TNFα, IL-1b, and IL-6 were obtained from Vector Best, Novosibirsk, Russia (ELISA kit cat. No. A-8756, A-8766, and A-8768). For TGFβ detection, we used the ELISA kit cat. no. SEA124Rb (“Cloude-Clone Corp.”, Katy, TX, USA). The optical density of the solutions in the wells of the strips was measured using a spectrophotometer (“Pikon”, Moscow, Russia) at 450 nm.

### 4.6. Statistical Analysis

The data represent the means ± standard error of means (SEM) from five to seven experiments. Statistical processing was performed using the Statistica 10.0 program. The normality of data distribution was tested using the Shapiro–Wilk test. Further processing was carried out using the Mann–Whitney method of non-parametric statistics for cytokine determination. Differences between groups were considered significant at *p* ≤ 0.05. The statistical significance for the PBMC viability was estimated using Student’s *t*-test, with *p* < 0.05 as the criterion of significance.

## 5. Conclusions

This study aimed to evaluate the effect of the progestins P4, MPA, and GB on the immune functions of PBMCs in menopausal women, showing a clear inhibition of their oxidative activity—an effect also inherent in NSAIDs.

A multidirectional effect of progestins on the production of cytokines, depending on their structure, was revealed: the synthetic progestins MPA and GB stimulated IL-6 and TNFα production, and P4 inhibited IL-6 and had no effect on TNFα. All the progestins had an anti-TGFβ action and tended to stimulate IL-1β.

The choice of progestin for menopausal women as a part of HRT or endometrium defense should be based on the age and hormonal status of the women, individual sensitivity (the expression and polymorphism of hormone receptor genes), and the nature of the concomitant inflammatory process (acute or chronic). Further research is required for the identification of the supranuclear targets of progesterone action, such as membrane progesterone receptors or other not-yet-identified structures.

## Figures and Tables

**Table 1:** Relative binding activity (RBA) of gestobutanoyl to progesterone receptors compared to other progestins.

Progestin	RBA, %	Reference and Comment
P4	100 *	[38,39]
MPA	298 *	[38,39]
Levonorgestrel	323 *	[38,39]
Dienogest	10 *	[38,39]
Drospirenon	19 *	[38,39]
Didrogesterone	85 *	[40]
Gestobutanoyl	4.5 **	[34], rabbit uterus tissue
	0.91 **	[34], rat uterus tissue
	59 **	[37], human endometrium tissue
	1700 **	[36], HeLa cancer cell line

Note: * values were determined using recombinant human receptor binding in vitro [39]; ** values were determined in a tissue by the radioligand binding assay as compared with P4 (100%).

## Data Availability

Not applicable.

## Abbreviations

GPX	Glutathione peroxidase
GST	Glutathione S Transferase
HRT	Hormone replacement therapy
LDC	Luminol-dependent chemiluminescence
LPS	lipopolysaccharide
MTT	3-(4,5-Dimethylthiazol-2-yl)-2,5-diphenytetrazolium bromide
NOS	Nitric oxide synthases
PBMC	Peripheral blood mononuclear cells
PHA	Phytohaemagglutinin
SOD	Superoxide dismutase

## References

[1] Piccinni M.P., Lombardelli L., Logiodice F., Kullolli O., Maggi E., Barkley M.S. (2019). Medroxyprogesterone Acetate Decreases Th1, Th17, and Increases Th22 Responses via AHR Signaling Which Could Affect Susceptibility to Infections and Inflammatory Disease. Front. Immunol..

[2] https://www.cancer.org/cancer/endometrial-cancer/treating/hormone-therapy.html.

[3] Fedotcheva T.A. (2021). Clinical Use of Progestins and Their Mechanisms of Action: Present and Future (Review). Sovrem. Tekhnologii Med..

[4] Petrosyan M.A., Balashova N.N., Polyanskikh L.S., Baziyan E.V., Tral’ T.G., Fasakhutdinova L.K., Razygraev A.V., Sapronov N.S. (2018). Influence of progesterone analogs on endometrioid heterotopia in experimental model of endometriosis. Exp. Clin. Pharmacol..

[5] Sergeev P.V., Semeĭkin A.V., Smirnova Z.S., Rzheznikov V.M., Grinenko G.S., Fedosov A.V., Fedotcheva T.A., Shimanovskiĭ N.L. (2004). Antitumor activity of the new gestagen 17alpha-acetoxy-3beta-butanoyloxy-6-methyl-pregna-4,6-dien-20-one. Exp. Clin. Pharmacol..

[6] Polikarpova A.V., Levina I.S., Sigai N.V., Zavarzin I.V., Morozov I.A., Rubtsov P.M., Guseva A.A., Smirnova O.V., Shchelkunova T.A. (2019). Immunomodulatory effects of progesterone and selective ligands of membrane progesterone receptors. Steroids.

[7] Marks M.A., Gravitt P.E., Burk R.D., Studentsov Y., Farzadegan H., Klein S.L. (2010). Progesterone and 17beta-estradiol enhance regulatory responses to human papillomavirus type 16 virus-like particles in peripheral blood mononuclear cells from healthy women. Clin. Vaccine Immunol..

[8] AbdulHussain G., Azizieh F., Makhseed M., Raghupathy R. (2020). Effects of Progesterone, Dydrogesterone and Estrogen on the Production of Th1/Th2/Th17 Cytokines by Lymphocytes from Women with Recurrent Spontaneous Miscarriage. J. Reprod. Immunol..

[9] Pfeilschifter J., Köditz R., Pfohl M., Schatz H. (2002). Changes in proinflammatory cytokine activity after menopause. Endocr. Rev..

[10] Yuan Y., Shimizu I., Shen M., Aoyagi E., Takenaka H., Itagaki T., Urata M., Sannomiya K., Kohno N., Tamaki K. (2008). Effects of estradiol and progesterone on the proinflammatory cytokine production by mononuclear cells from patients with chronic hepatitis C. World J. Gastroenterol..

[11] Straub R.H. (2007). The complex role of estrogens in inflammation. Endocr. Rev..

[12] Fedotcheva T.A., Sheichenko O.P., Fedotcheva N.I. (2021). New Properties and Mitochondrial Targets of Polyphenol Agrimoniin as a Natural Anticancer and Preventive. Agent Pharm..

[13] Maeda Y., Ohtsuka H., Tomioka M., Tanabe T., Nambo Y., Uematsu H., Oikawa M. (2012). Effect of progesterone on the in vitro response of peripheral blood mononuclear cells stimulated by Escherichia coli in mares. J. Vet. Med. Sci..

[14] Huber N., Marasco V., Painer J., Vetter S.G., Göritz F., Kaczensky P., Walzer C. (2019). Leukocyte Coping Capacity: An Integrative Parameter for Wildlife Welfare Within Conservation Interventions. Front. Vet. Sci..

[15] Chausova S.V., Gurevich K.G., Bondareva G.P., Filatov O.J., Malyshev I.Y. (2015). The role of cellular mediators in the development of the phenomenon of inhibition induced by barium sulfate luminol-dependent chemiluminescence of blood under the influence of non-steroidal anti-inflammatory drugs in patients with intolerance to these drugs. Patol. Fiziol. I Eksperimental’naia Ter..

[16] Marren K. (2011). Dimethyl sulfoxide: An effective penetration enhancer for topical administration of NSAIDs. Phys. Sportsmed..

[17] (2005). Drugbank. https://go.drugbank.com/drugs/DB01093.

[18] Ivkin D.Y., Okovitiy S.V., Ivkina A.S., Anisimova N.A. (2019). Dimetilsulfoxid-veschestvo s pleyotropnyimi effektami, aktual’nyimi pri zabolevaniyah oporno-dvigatel’nogo apparata. Lechaschiy Vrach.

[19] Hughes D.L., Richards R.S., Lexis L.A. (2018). Using chemiluminescence to determine whole blood antioxidant capacity in rheumatoid arthritis and Parkinson’s disease patients. Luminescence.

[20] Costa D., Moutinho L., Lima J.L.F.C., Fernandes E. (2006). Antioxidant activity and inhibition of human neutrophil oxidative burst mediated by arylpropionic acid non-steroidal anti-inflammatory drugs. Biol. Pharm. Bull..

[21] Raghupathy R., Al-Azemi M. (2015). Modulation of Cytokine Production by the Dydrogesterone Metabolite Dihydrodydrogesterone. Am. J. Reprod. Immunol..

[22] Raghupathy R., Mutawa E.A., Makhseed M., Al-Azemi M., Azizieh F. (2007). Redirection of cytokine production by lymphocytes from women with pre-term delivery by dydrogesterone. Am. J. Reprod. Immunol..

[23] Hantoushzadeh S., Aliabad R.A., Norooznezhad A.H. (2020). Antibiotics, Inflammation, and Preterm Labor: A Missed Conclusion. J. Inflamm. Res..

[24] Mantovani G., Macciò A., Lai P., Massa E., Ghiani M., Santona M.C. (1998). Cytokine involvement in cancer anorexia/cachexia: Role of megestrol acetate and medroxyprogesterone acetate on cytokine downregulation and improvement of clinical symptoms. Crit. Rev. Oncog..

[25] Kurebayashi J., Yamamoto S., Otsuki T., Sonoo H. (1999). Medroxyprogesterone acetate inhibits interleukin 6 secretion from KPL-4 human breast cancer cells both in vitro and in vivo: A possible mechanism of the anticachectic effect. Br. J. Cancer.

[26] Groh L.A., Verel D.E., van der Heijden C.D.C.C., Matzaraki V., Moorlag S.J.C.F.M., de Bree L.C., Koeken V.A.C.M., Mourits V.P., Keating S.T., van Puffelen J.H. (2022). Immune modulatory effects of progesterone on oxLDL-induced trained immunity in monocytes. J. Leukoc. Biol..

[27] Brundin P.M.A., Landgren B.-M., Fjällström P., Shamekh M.M., Gustafsson J.-A., Johansson A.F., Nalvarte I. (2021). Expression of Sex Hormone Receptor and Immune Response Genes in Peripheral Blood Mononuclear Cells during the Menstrual Cycle. Front. Endocrinol..

[28] Fedotcheva T.A., Fedotcheva N.I., Shimanovsky N.L. (2022). Progesterone as an Anti-Inflammatory Drug and Immunomodulator: New Aspects in Hormonal Regulation of the Inflammation. Biomolecules.

[29] Koubovec D., Ronacher K., Stubsrud E., Louw A., Hapgood J.P. (2005). Synthetic progestins used in HRT have different glucocorticoid agonist properties. Mol. Cell Endocrinol..

[30] Ozdemir F., Ovali E., Aydin F., Kavgaci H., Büyükcelik A., Ucar F., Sönmez M. (2004). The in-vitro effects of medroxyprogesterone acetate on acidic pH induced apoptosis of periferal blood mononuclear cells. J. Exp. Clin. Cancer Res..

[31] Hardy D.B., Janowski B.A., Corey D.R., Mendelson C.R. (2006). Progesterone receptor plays a major antiinflammatory role in human myometrial cells by antagonism of nuclear factor-kappaB activation of cyclooxygenase 2 expression. Mol. Endocrinol..

[32] Miller L., Hunt J.S. (1998). Regulation of TNF-alpha production in activated mouse macrophages by progesterone. J. Immunol..

[33] Africander D., Verhoog N., Hapgood J.P. (2011). Molecular mechanisms of steroid receptor-mediated actions by synthetic progestins used in HRT and contraception. Steroids.

[34] Zeynalov O.A., Savinova T.S., Andryushina V.A., Petrosyan M.A. (2018). Synthetic analogues of progesterone in in vitro and in vivo models. Biomedicine.

[35] Liga A.B., Ukhina T.V., Shimanovskii N.L. (2008). Activity lysosomal enzymes in rat skin fibroblasts after treatment with progesterone and new gestagen ABMP. Bull. Exp. Biol. Med..

[36] Sergeev P.V., Fedotcheva T.A., Rzheznikov V.M., Grinenko G.S., Semeĭkin A.V., Vetchinkina V.B., Atroshkin K.A., Shimanovskiĭ N.L. (2007). A new Russian gestagen with anticancer activity. Vestn. Ross. Akad. Med. Nauk.

[37] Kareva E.N., Grinenko G.S., Gasparian N.D., Ovchinnikova E.V., Gorenkova O.S. (2006). Influence of the structure of synthetic gestagens on their binding to progesteron receptors in the endometrium. Eksp. Klin. Farm..

[38] Sitruk-Ware R. (2008). Pharmacological profile of progestins. Maturitas.

[39] Stanczyk F.Z., Hapgood J.P., Winer S., Mishell D.R. (2013). Progestogens used in postmenopausal hormone therapy: Differences in their pharmacological properties, intracellular actions, and clinical effects. Endocr. Rev..

[40] Schindler A.E. (2009). Progestational effects of dydrogesterone in vitro, in vivo and on the human endometrium. Maturitas.

[41] Zeinalov O.A., Andryushina V.A., Yaderets V.V. (2022). New synthetic analogs of progesterone: From the search for an active molecule to clinical use (review of our own research). Pharm. Chem. J..

[42] Africander D., Louw R., Verhoog N., Noeth D., Hapgood J.P. (2011). Differential regulation of endogenous pro-inflammatory cytokine genes by medroxyprogesterone acetate and norethisterone acetate in cell lines of the female genital tract. Contraception.

[43] Proskurnina E.V., Polimova A.M., Sozarukova M.M., Vladimirov Y.A., Prudnikova M.A., Ametov A.S. (2016). Kinetic chemiluminescence as a method for oxidative stress evaluation in examinations of patients with type 2 diabetes mellitus. Bull. Exp. Biol. Med..

[44] Gaudio E., Bordin S., Lora I., Lora M., Massignani M., Benedictis G.M.D. (2018). Leukocyte coping capacity chemiluminescence as an innovative tool for stress and pain assessment in calves undergoing ring castration. J. Anim. Sci..

[45] Nascimento C.R., Fernandes N.A.R., Maldonado L.A.G., Junior C.R. (2022). Comparison of monocytic cell lines U937 and THP-1 as macrophage models for in vitro studies. Biochem. Biophys. Rep..

[46] Hall O.J., Klein S.L. (2017). Progesterone-based compounds affect immune responses and susceptibility to infections at diverse mucosal sites. Mucosal. Immunol..

[47] Mantovani G., Macciò A., Esu S., Lai P., Santona M.C., Massa E., Dessì D., Melis G.B., Del Giacco G.S. (1997). Medroxyprogesterone acetate reduces the in vitro production of cytokines and serotonin involved in anorexia/cachexia and emesis by peripheral blood mononuclear cells of cancer patients. Eur. J. Cancer.

[48] Huijbregts R.P., Helton E.S., Michel K.G., Sabbaj S., Richter H.E., Goepfert P.A., Hel Z. (2013). Hormonal contraception and HIV-1 infection: Medroxyprogesterone acetate suppresses innate and adaptive immune mechanisms. Endocrinology.

[49] García-Juárez M., Camacho-Morales A. (2022). Defining the Role of Anti- and Pro-inflammatory Outcomes of Interleukin-6 in Mental Health. Neuroscience.

[50] Askari V.R., Rahimi V.B., Zargarani R., Ghodsi R., Boskabady M., Boskabady M.H. (2021). Anti-oxidant and anti-inflammatory effects of auraptene on phytohemagglutinin (PHA)-induced inflammation in human lymphocytes. Pharmacol. Rep..

[51] Komane M., Avenant C., Louw-du Toit R., Africander D.J., Hapgood J.P. (2022). Differential off-target glucocorticoid activity of progestins used in endocrine therapy. Steroids.

[52] Preciado-Martínez E., García-Ruíz G., Flores-Espinosa P., Bermejo-Martínez L., Espejel-Nuñez A., Estrada-Gutiérrez G., Razo-Aguilera G., Granados-Cepeda M., Helguera-Repetto A.C., Irles C. (2018). Progesterone suppresses the lipopolysaccharide-induced pro-inflammatory response in primary mononuclear cells isolated from human placental blood. Immunol. Investig..

[53] Garcia-Ruíz G., Flores-Espinosa P., Preciado-Martínez E., Bermejo-Martínez L., Espejel-Nuñez A., Estrada-Gutierrez G., Maida-Claros R., Flores-Pliego A., Zaga-Clavellina V. (2015). In vitro progesterone modulation on bacterial endotoxin-induced production of IL-1β, TNF-α, IL-6, IL-8, IL-10, MIP-1α, and MMP-9 in pre-labor human term placenta. Reprod. Biol. Endocrinol..

[54] Bouman A., Schipper M., Heineman M.J., Faas M. (2004). 17beta-estradiol and progesterone do not influence the production of cytokines from lipopolysaccharide-stimulated monocytes in humans. Fertil. Steril..

[55] Jain S.K., Kannan K., Prouty L., Jain S.K. (2004). Progesterone, but not 17beta-estradiol, increases TNF-alpha secretion in U937 monocytes. Cytokine.

[56] Campe K.-N.J., Redlich A., Zenclussen A.C., Busse M. (2022). An increased proportion of progesterone receptor A in peripheral B cells from women who ultimately underwent spontaneous preterm birth. J. Reprod. Immunol..

[57] Hudić I., Szekeres-Bartho J., Fatušić Z., Stray-Pedersen B., Dizdarević-Hudić L., Latifagić A., Hotić N., Kamerić L., Mandžić A. (2011). Dydrogesterone supplementation in women with threatened preterm delivery--the impact on cytokine profile, hormone profile, and progesterone-induced blocking factor. J. Reprod. Immunol..

[58] Konecna L., Yan M.S., Miller L.E., Schölmerich J., Falk W., Straub R.H. (2000). Modulation of IL-6 production during the menstrual cycle in vivo and in vitro. Brain Behav. Immun..

[59] Sciarra F., Campolo F., Franceschini E., Carlomagno F., Venneri M.A. (2023). Gender-Specific Impact of Sex Hormones on the Immune System. Int. J. Mol. Sci..

[60] Stefanaki C., Bacopoulou F., Chrousos G.P. (2022). Gut Microsex/Genderome, Immunity and the Stress Response in the Sexes: An Updated Review. Sexes.

[61] Lissauer D., Eldershaw S.A., Inman C.F., Coomarasamy A., Moss P.A., Kilby M.D. (2015). Progesterone promotes maternal-fetal tolerance by reducing human maternal T-cell polyfunctionality and inducing a specific cytokine profile. Eur. J. Immunol..

[62] Omu A.E., Al-Azemi M.K., Makhseed M., Al-Oattan F., Ismail A.A., Al-Tahir S., Al-Busiri N. (2003). Differential expression of T-helper cytokines in the peritoneal fluid of women with normal ovarian cycle compared with women with chronic anovulation. Acta Obstet. Gynecol. Scand..

[63] Baysal B. (2001). Comparison of the resorbable barrier interceed (TC7) and preoperative use of medroxyprogesterone acetate in postoperative adhesion prevention. Clin. Exp. Obstet. Gynecol..

[64] Böyum A. (1968). Isolation of mononuclear cells and granulocytes from human blood. Isolation of mononuclear cells by one centrifugation, and of granulocytes by combining centrifugation and sedimentation at 1 g. Scand. J. Clin. Lab. Investig. Suppl..

